# Systematic review of illness uncertainty management interventions for cancer patients and their family caregivers

**DOI:** 10.1007/s00520-020-05931-x

**Published:** 2021-01-25

**Authors:** Ting Guan, Yousef Qan’ir, Lixin Song

**Affiliations:** 1 School of Social Work, University of North Carolina at Chapel Hill, Chapel Hill, NC USA; 2 School of Nursing, University of North Carolina at Chapel Hill, Chapel Hill, NC USA; 3 Lineberger Comprehensive Cancer Center, University of North Carolina at Chapel Hill, Chapel Hill, NC USA

**Keywords:** Cancer, Illness uncertainty, Intervention, Family caregiver, Systematic review, Social support

## Abstract

**Purpose:**

Illness uncertainty pervades individuals’ experiences of cancer across the illness trajectory and is associated with poor psychological adjustment. This review systematically examined the characteristics and outcomes of interventions promoting illness uncertainty management among cancer patients and/or their family caregivers.

**Methods:**

PubMed, Scopus, PsycINFO, Cumulative Index to Nursing and Allied Health Literature (CINAHL), Embase, and Cochrane Database of Systematic Reviews were systematically searched for relevant literature. We included randomized controlled trials (RCTs) and quasi-experimental studies focusing on interventions for uncertainty management in cancer patients and/or their family caregivers.

**Results:**

Our database searches yielded 26 studies. Twenty interventions were only offered to cancer patients, who were mostly elder, female, and White. All interventions included informational support. Other intervention components included emotional support, appraisal support, and instrumental support. Most interventions were delivered in person and via telephone (*n* = 8) or exclusively in person (*n* = 7). Overall, 18 studies identified positive intervention effects on illness uncertainty outcomes.

**Conclusion:**

This systematic review foregrounds the promising potential of several interventions—and especially multi-component interventions—to promote uncertainty management among cancer patients and their family caregivers. To further improve these interventions’ effectiveness and expand their potential impact, future uncertainty management interventions should be tested among more diverse populations using rigorous methodologies.

## Introduction

*Illness uncertainty* is defined as “the inability of a person to determine the meaning of illness-related events” [1]. It can persist across the cancer trajectory from the time of diagnosis, through treatment, to long-term survivorship [2] and can be exacerbated by disease progression [3]. Illness uncertainty is widely recognized as a common and significant source of psychosocial stress among cancer patients [4], and studies have shown that increased uncertainty adversely affects cancer patients’ psychological adjustment [5, 6], health behaviors [7], and quality of life [8, 9]. This uncertainty can also extend to cancer patients’ family caregivers. In fact, patients’ partners have often reported higher levels of uncertainty compared to patients [3]. Research has also shown that increases in family caregivers’ illness uncertainty are associated with poorer psychological adjustment to the diagnoses and progression of cancer in patients [10, 11]. For example, uncertainty about the unknown outcomes of childhood cancers (e.g., late effects of cancer treatment, relapse) can increase parents’ distress and dysfunctional behaviors [10].

To address the negative impacts of illness uncertainty on the health outcomes (e.g., quality of life) [12], researchers and practitioners have developed and implemented various interventions to help cancer patients and their family caregivers manage illness uncertainty. Three previous literature reviews have synthesized research developments related to uncertainty management interventions. In their review of interventions for managing uncertainty and fear of recurrence in female breast cancer survivors [13], Dawson and colleagues reported that the main intervention components included mindfulness, more effective patient–provider communication, and stress management through counseling [13]. In their integrative literature review of uncertainty among children with chronic illnesses and their families, Gunter and Duke concluded that the education and psychosocial support is important in reducing uncertainty [14]. In their recent meta-analysis of psychosocial uncertainty management interventions among adult patients with various diagnoses (e.g., cancer, HIV, heart disease) and their family caregivers [15], Zhang et al. reported that psychosocial interventions are effective in reducing short- and long-term uncertainty both among patients and their family caregivers [15].

The existing reviews have focused on patients with various types of chronic illnesses who may face different challenges from patients with cancer [16] or patients with a gender-specific type of cancer (e.g., breast cancer). It therefore remains unclear whether the findings of these reviews are generalizable to patients with other types of cancer. Additionally, although research has shown that children and adolescents with cancer are affected by illness uncertainty [17, 18], no systematic review has examined their experiences of uncertainty management interventions. Researchers and practitioners stand to benefit from a comprehensive review of the literature about illness uncertainty interventions for patients with different types of cancer across age groups and their family caregivers. To this end, our study (a) systematically reviews and synthesizes results of uncertainty management interventions for cancer patients and their family caregivers, (b) identifies the strengths and gaps in this line of research, and (c) suggests directions for future research. Specifically, our systematic review examines the characteristics of participants in studies of illness uncertainty management interventions as well as the characteristics and outcomes of those interventions.

## Methods

We adapted a comprehensive systematic review protocol based on the Cochrane Collaboration and the Preferred Reporting Items for Systematic Reviews and Meta-Analyses (PRISMA) guidelines [19]. This protocol was registered with PROSPERO, an international prospective register of systematic reviews, prior to the beginning of the study (registration number CRD42019128004).

### Eligibility criteria

We used the population, interventions, comparator, outcomes, and study (PICOS) design(s) to guide our inclusion criteria [20]. Studies eligible for inclusion are as follows: (a) targeted cancer patients and/or their family caregivers; (b) included uncertainty management in their research aims and/or as a part of the intervention’s contents; (c) reported intervention effects on illness uncertainty; (d) used randomized controlled trials (RCTs) or quasi-experimental designs; and (e) were published in English between January 1, 2000 and December 31, 2019. The search was not limited to studies using a control or comparison group.

### Search methods

A university health sciences librarian helped to develop the search terms and identify relevant search databases. We conducted electronic literature searches using six databases: PubMed, Scopus, PsycINFO, Cumulative Index to Nursing and Allied Health Literature (CINAHL), Embase, and Cochrane Database of Systematic Reviews. The database searches used Boolean terms “OR” and “AND” with combinations of the following search terms: (uncertainty) AND (cancer OR neoplasm*OR tumor OR myeloma OR oncolog*) AND (intervention OR program OR effect OR effectiveness OR treatment OR therapy) AND (patient OR patients OR caregiv* OR family OR families) AND (psych* OR mental* OR emotion*).

To identify studies potentially overlooked by our electronic searches, our research team conducted forward and backward citation chaining and hand searched Web of Science, Google Scholar, and prominent journals in the field to identify relevant articles for inclusion. Two coauthors independently reviewed the titles and abstracts and then—if an article merited further consideration—its full text using Covidence. Covidence is a web-based software platform designed to support the efficient production of systematic reviews [21]. We resolved any discrepancies in the two coauthors’ respective decisions regarding articles’ inclusion via group discussion among all team members.

### Assessment of risk of bias in the included studies

We used the Cochrane Collaboration’s Risk of Bias Tool [22] to assess various sources of bias: selection bias, performance bias, detection bias, attrition bias, reporting bias, and other possible sources of bias (Appendix). Each domain was endorsed with a rating of “low risk,” “high risk,” or “unclear risk” following guideline’s criteria. Two coauthors independently conducted all risk of bias assessments, and we resolved any differences in their assessments through team discussion.

### Data extraction and synthesis

Two of the coauthors independently extracted relevant data from the studies that met our inclusion criteria. We compared these extracted results and resolved any discrepancies through team discussion before merging the data. Because the included studies displayed different participant characteristics, intervention components, outcomes, and follow-up periods, we could not conduct a meta-analysis of their findings. We summarized the narratives and themes of each study and its results. Guided by House’s conceptualization of social support [23], we classified each intervention’s components into four categories: informational support, emotional support, appraisal support, and instrumental support.

## Results

**Fig. 1:**
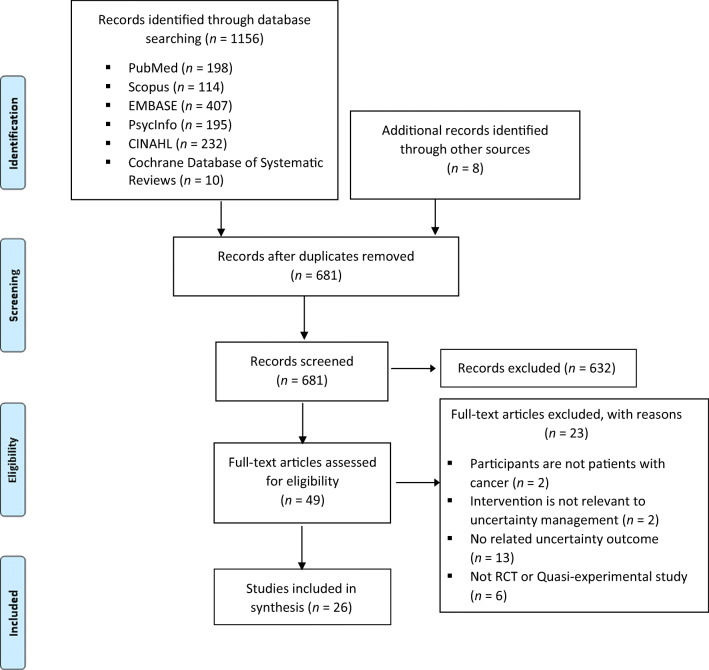
PRISMA flow diagram

As shown in Fig. 1, our initial search of electronic databases and records and our hand searches of other sources yielded 1156 records. After removing duplicates, we identified 681 articles for title and abstract review, of which 49 were retained for a full-text review. After removing the studies that did not meet the inclusion criteria, we included 26 articles in this review.

### Characteristics of studies

The majority of studies were conducted in the USA (*n* = 16). Others were conducted in Canada, China, Japan, United Kingdom (UK), and Vietnam. Eighteen studies were RCTs; one study used a RCT crossover design [24]. Eight studies were quasi-experimental studies (i.e., five “1-group pretest-posttest” studies, two “2-group pretest-posttest” studies, and one “2-group posttest” study) (Table 1). Among the 20 studies that included a control group, 12 studies used usual care, and eight included an active control group (e.g., a self-help group [25]; groups receiving recorded and written messages [26], telephone calls [27], and delayed interventions [28]). Sample sizes of included studies ranged from 9 [29] to 968 participants [30]. Among all studies, only seven studies were guided by theoretical frameworks such as the stress and coping theory (*n* = 5) [30–34], the uncertainty in illness theory (*n* = 1) [29], the double ABCX model (*n* = 1) [34], and the resilience model (*n* = 1) [35].

**Table 1:** Characteristics of study and participants and uncertainty outcomes

Lead author, year, and country	Study characteristics			Participant characteristics				Uncertainty measures		Results
	Theoretical basis	Design	Sample size (I and C)^[1]^	Cancer type and stage of survivorship	Mean age (years)	Gender	Race	Measurement	Assessment time point	
Chow, 2014, China	N/A	2-arm RCT	26 (13, 13)	Patients newly diagnosed with gynecological cancer	I: 51.4 C: 57.7	100% female	N/A	MUIS	3 (BL, end of intervention, 8-week post-intervention)	More reduction in the Inconsistency subscale at the end of intervention in the intervention group (*p* = 0.026). No significant group difference at 8 weeks.
Christman, 2004, USA	N/A	3-arm RCT	76 (25, 26, 26)	Patients with mixed cancer receiving radiation therapy	55	68% female	92% Caucasian	MUIS-symptom subscale	2 (1 day after each intervention)	Less uncertainty in the concrete objective information intervention group (*p* < 0.05). No result reported about the difference between elaxation instruction intervention group and control groups.
Dharmarajan, 2019, USA	N/A	1-arm quasi-experimental	40	Patients with advanced mixed cancer	N/A	57% female	75% White 15% Black 8% Hispanic 2% Asian	DCS-U	2 (BL, post-intervention)	Significant decrease over time (*p* = 0.02).
El-Jawahri, 2010, USA	N/A	2-arm RCT	50 (23, 27)	Patients with brain tumor with a poor prognosis	54	56% male	92% White	DCS-U	2 (BL, post-intervention)	More reduction in uncertainty in the intervention group (*p* = 0.002).
Germino, 2013, USA	N/A	2-arm RCT	313 (167, 146)	Breast cancer survivors	44	100% female	63% Caucasian 37% African American	MUIS-S	3 (BL, 4–6 and 8–10-month post-BL)	Greater decrease in uncertainty in the intervention group over time (*p* = 0.01).
Ha, 2019, Vietnam	N/A	2-arm quasi-experimental	115 (57, 58)	Patients with breast cancer receiving mastectomy	N/A	100% female	N/A	MUIS-Short Form	1 (1 week after mastectomy)	More reduction in uncertainty in the intervention group (*p* < 0.05).
Hendricks-Ferguson, 2017, USA	Stress-coping theory; double ABCX model	1-arm quasi-experimental	13	Parents of hospital children diagnosis with brain tumor and a poor prognosis	N/A	85.7% female	69.2% Caucasian 15.4% African American 15.4% Hispanic	Parent Experience of Child Illness-Short Form	4 (BL, after session 1, session 2, and session 3)	Significant decrease over time (*p* = 0.0432).
Kazer, 2011, USA	Uncertainty in illness theory	1-arm quasi-experimental	9	Patients with prostate cancer and undergoing active surveillance	72	100% male	100% Caucasian	MUIS-C	3 (BL, 5 and 10-week post-BL)	No difference over time.
Lebel, 2014, Canada	N/A	1-arm quasi-experimental	56	Patients with mixed cancer and after treatment	54.8	100% female	80.8% Caucasian 10.6% Asian 4.3% Hispanic 4.3% others	MUIS	3 (pre-intervention, post-intervention, at 3-month post-intervention)	Significant decrease over time (*p* < 0.001).
Liu, 2006, China	N/A	2-arm quasi-experimental	61 (31, 30)	Patients newly diagnosed with breast cancer	I: 48.1 C: 46.6	100% female	N/A	MUIS	3 (BL, 1 and 3 months after surgery)	More reduction in uncertainty in the intervention at 3 months after surgery (*p* < 0.01).
McCaughan, 2018, UK	Stress-coping theory	2-arm RCT	17 dyads (13, 4)	Patients with prostate cancer and post-treatment and their spouse/partner	I: patient: 67.5 Partner: 64.1 C: Patient: 63.8 Partner: 60	Patient: 100% male Spouse: 100% female	100% Caucasian	MUIS	3 (BL, post-intervention, 1-month post-intervention)	No group difference.
McCorkle, 2009, USA	N/A	2-arm RCT	123 (63, 60)	Patients with ovarian cancer and prognosis of at least 6 months	I: 58.4 C: 62.2	100% female	I: 90.5% White C: 93.3% White	MUIS-ambiguity subscale	4 (BL, 1, 3, and 6 months post-surgery)	Greater improvement in the ambiguity subscale in the intervention group over time (*p* = 0.0001).
Mishel, 2002, USA	N/A	3-arm RCT	239	Patients with prostate cancer and after treatment	64.0	100% male	56% Caucasian 44% African American	MUIS	3 (BL, 4 and 7 months post-BL)	No group difference.
Mishel, 2009, USA	N/A	3-arm RCT	256 (93,89, 74)	Patients with prostate cancer and before the treatment	62.5	100% male	71.5% Caucasian 28.5% African American	Problem-solving, patient–provider communication, and cancer knowledge	3 (BL, 4 weeks and 3 months of post-BL)	Greater improvement in cancer knowledge (*p* = 0.0001), problem-solving (*p* = 0.05), and patient–provider communication (*p* = 0.01) for intervention group over time.
Mori, 2019, Japan	N/A	Crossover RCT	105	Patients with breast cancer recurrence	53.8	100% female	N/A	1-item scale	4 (after each video)	Lower uncertainty in the group viewing the video with more versus less explicit disclosure (*p* = 0.032)
Northouse, 2005, USA	Stress-coping theory	2-arm RCT	134 dyads (69,65)	Patients with breast cancer and recurrent within the previous month and their spouses	Patient: 54 Partner: 52	Patient: 100% female Spouse: 100% male	77% Caucasian 19% African American 4% Hispanic, Asian, or Native American	MUIS	3 (BL, 3 and 6 months post-BL)	No group difference.
Northouse, 2007, USA	Stress-coping theory	2-arm RCT	263 dyads (129, 134)	Patients with newly diagnosed, recurrence, and advanced prostate cancer and their spouses	Patient: 63 Spouse: 59	Patient: 100% male Spouse: 100% female	84% Caucasian 14% African American 2% Hispanic, Asian, Native American, or mixed race	MUIS	4 (BL, 4, 8, and 12 months post-BL)	Less uncertainty in the intervention patients at 4 months (*p* < 0.05) and in the intervention spouses at 4 months *(p* < 0.01) and 8 months (*p* < 0.05).
Northouse, 2013, USA	Stress-coping theory	3-arm RCT	484 dyads (159, 162, 163)	Patients with mixed cancer and newly diagnosed with advanced cancer and their caregiver	Patient: 60.5 Family caregiver: 56.7	Patient: 61.4% female Family caregiver: 55.8% female	82.5% Caucasian 13.5% African American 1% Hispanic 1.3% American Indian 1.3% Asian 0.3% multi-racial	MUIS	3 (BL, 3 and 6 months post-BL)	No group difference.
Ritz, 2000, USA	N/A	2-arm RCT	210 (106, 104)	Patients with newly diagnosed breast cancer	I: 55.7 C: 55.3	100% female	I: 97% White, 2% Asian, 1% African American C: 97% White, 1% Asian, 1% African American, 1% American Indian	MUIS	6 (1, 3, 6, 12, 18, and 24 months)	Less uncertainty in the intervention group at 1 month (*p* = 0.001), 3 months (*p* = 0.026), and 6 months (*p* = 0.011).
Schulman-Green, 2017, USA	N/A	1-arm quasi-experimental	105	Patients with breast cancer and had a prognosis of at least 3 months	52.3	100% female	78.1% White 9.5% Black 5.7% Hispanic 6.7% other	MUIS	2 (BL and post-intervention)	No difference over time.
Sussman, 2018, Canada	N/A	2-arm RCT	193 (89, 104)	Patients with newly diagnosed mixed cancer	I: 61 C: 60	I: 84% female C: 76% female	N/A	MUIS-C	3 (BL, 2‑3 weeks and 8‑10 weeks of post-BL)	No group difference.
Tomei, 2018, Canada	N/A	2-arm RCT	25 (11, 14)	Patients with mixed cancer and after treatment	55	100% female	95.8% Caucasian 4.2% Asian	MUIS-C	3 (pre-intervention, post-intervention, at 3-month post-intervention)	Greater improvements in uncertainty in the intervention group over time (*p* = 0.002).
Victorson, 2017, USA	N/A	2-arm RCT	43 (24, 19)	Patients diagnosed with low-risk localized prostate cancer on active surveillance	I: 71.2 C: 69.4	100% male	I: 94.44% Caucasian, 5.56% African American C: 95.65% Caucasian, 4.35% African American	IUS-Short Form	4 (BL, 8 weeks, 6 months, and 12 months of post-BL)	No group difference.
Wang, 2018, China	N/A	2-arm quasi-experimental	101 (51, 50)	Parents of hospital children newly diagnosed with acute lymphoblastic leukemia	N/A	I: 67% female C: 77% female	I: 98% Han nationality, 2% ethnic minority C: 100% Han nationality	PPUS	2 (BL and 3 months post-BL)	Less uncertainty in the intervention group at 3 months (*p* = 0.01).
Wells-Di Gregorio, 2019, USA	N/A	2-arm RCT	28 (17, 11)	Patients with advanced mixed cancer	56.54	82% female	93% Caucasian, 7% African American	IUS	2 (BL and 6 weeks post-intervention)	No group difference.
Ye, 2016, China	Resilience model	2-arm RCT	204 (101, 103)^[2]^	Patients with breast cancer and after treatment	N/A	100% female	I: 95.7% Han nationality, 4.3% ethnic minority C: 93.9% Han nationality, 6.1% ethnic minority	MUIS-Short Form	4 (BL, 2 months, 6 months, and 12 months of post-BL)	Lower uncertainty in the intervention group over time (*p* < 0.01).

^[1]^ Sample size is individual unless defined otherwise. ^[2]^ The study also included a group of women without breast cancer. However we only focused on the patients with breast cancer and those in the control group. *I*, intervention; *C*, control; *N/A*, data not available; *BL*, baseline; *RCT*, randomized controlled trial; *MUIS*, Mishel’s Uncertainty in Illness Scale including four subscales: ambiguity, complexity, inconsistency, and unpredictability; *MUIS-C*, Mishel Uncertainty in Illness Scale–Community Form; *MUIS-S*, Mishel Uncertainty in Illness Scale-Survivor version; *PPUS*, Parents’ Perception of Uncertainty Scale; *IUS*, Intolerance of Uncertainty Scale; *DCS-U*, Decisional Conflict Scale-uncertainty subscale

### Characteristics of participants

Twenty interventions were only offered to cancer patients. Two studies targeted family caregivers (i.e., the parents of children with cancer) [34, 36]. Four interventions were offered to both cancer patients and their partners and/or family caregivers [30–33].

Participants were recruited from hospitals, by invitation from a care provider, or via mailing and poster initiatives. The majority of studies recruited homogeneous patient populations, including patients with breast cancer (*n* = 8), prostate cancer (*n* = 6), brain tumor (*n* = 2), leukemia (*n* = 1), gynecological cancer (*n* = 1), and ovarian cancer (*n* = 1). Approximately 26.9% of studies (*n* = 7) targeted patients with various types of cancer. Regarding the stages of the cancer trajectory, these studies focused on cancer patients who were post-treatment (*n* = 8); patients with newly diagnosed cancer (*n* = 6); patients with advanced cancer (*n* = 5); patients undergoing active surveillance for cancer (*n* = 2); patients receiving treatment (*n* = 2); patients in recurrence (*n* = 2); and/or patients at a mix of stages (*n* = 1). With only two studies targeting parents of children and adolescents with cancer, the majority of the studies have focused on participants who were mostly female and White, and with an average age ranged from 44 to 72 years.

### Intervention characteristics

Table 2 summarizes the interventions’ characteristics. Nineteen studies (73.1%) included uncertainty management as their main aim.

**Table 2:** Characteristics of the interventions

Lead author and year	Study aim	Intervention				Control
		Theoretical basis	Component	Mode, format, duration, dosage	Interventionist	
Chow 2014	Test the feasibility of a psychoeducational intervention program^a^	Thematic counseling model	Information about cancer diagnosis, treatment, side effects, communication skills, behavior therapy (e.g., deep breathing), psychological support	In-person, individual + group, 8 weeks, 4 sessions (30–60 min)	Nurse	Contact after the operation and invite to join a self-help group
Christman 2004	Examine the effects of concrete objective information and relaxation effect	Self-regulation theory and varied relaxation strategies	Information provision about symptom management, and relaxation	Audiotape + booklet, individual, N/A, 2 sessions	N/A^b^	Professionally recorded and written messages
Dharmarajan 2019	Test the ability of a newly created video decision aid intervention effect^a^	N/A	Information provision about palliative radiation therapy, process, side effects	Video, individual, N/A, N/A	Palliative care physician involved in video content	N/A
El-Jawahri 2010	Determine the effect of use of goal-of-care video to improve end-of-life decision-making^a^	N/A	Information about medical care	Video, individual, 6-min video presentation, N/A	Oncologists, critical care intensivists, palliative care physician and medical ethics experts involved in video content	Verbal narrative
Germino 2013	Determine the effect of an uncertainty management intervention^a^	Theory of Uncertainty in Illness	Information about cognitive and behavior strategies, side effects, and resources	CD, individual, 4 weeks, 4 weekly sessions (20 min)	Nurse	Four 20-min phone calls from psychology graduate students
Ha 2019	Examined the effect of the uncertainty management program^a^	Theory of Uncertainty in Illness	Information provision, qigong practice, emotional disclosure skills, breathing relaxing, nutrition care, ongoing communication with nurses	In-person + telephone, individual, 3 weeks, 3 in-person sessions + 2 phone follow-ups	Nurse	Usual care
Hendricks-Ferguson 2017	Report feasibility, acceptability, and outcome of palliative and end-of-life communication intervention study^a^	N/A	Discussion about child’s disease status, prognosis, and treatment options following diagnosis to enhance hope and nonabandonment	In-person, family, 26 weeks, 3 sessions (time varied)	Neuro-oncology doctor and nurse	N/A
Kazer 2011	Provide preliminary data on an internet intervention^a^	N/A	Information about illness, cognitive reframe strategies, self-care management strategies, and life issues	Internet, individual, 5 weeks, N/A	Nurse	N/A
Lebel 2014	Develop, manualize, and pilot test the feasibility and preliminary efficacy of cognitive-existential group intervention	Leventhal’s common sense model, uncertainty in illness theory, cognitive models of worry	Introduction about illness, cognitive restructuring and triggers, coping skills (e.g., relaxation, calming self-talk, guided imagery); emotion expression, and specific fears confrontation	In-person, group, 6 weeks, 6 sessions (90 min)	Health care professionals with formal training in psychotherapy (psychologists, social workers, and nurses)	N/A
Liu 2006	Examine the effects of continuing supportive care intervention study^a^	N/A	Information, emotional and psychological support, referral and continual follow-up	In-person + telephone, individual, 3 months, 4 sessions (90, 30, 60, 15 min, respectively)	Nurse	Usual care
McCaughan 2018	Evaluate the process and outcome of a psychosocial intervention^a^	Theory of self-efficacy and theory of stress and coping	Information about disease and treatments	Telephone + in-person, group + family, 9 weeks, 3 group sessions (180 min) + 2 telephone sessions	Professionals specifically trained in the intervention	Usual care
McCorkle 2009	Examine the effect of a nursing intervention on quality of life^a^	N/A	Symptom management and monitoring, emotional support, patient education, care coordination of resources, referrals, and direct nursing care	In-person, individual, 6 months, 18 contacts (tailored to each patient’s need)	Nurse	Symptom management toolkit
Mishel 2002	Test the efficacy of an individualized uncertainty management intervention^a^	Theory of Uncertainty in Illness	Information about resources and skills to address problem; cognitive reframing	Telephone, individual + family, 8 weeks, 8 telephone calls	Nurse	Usual care
Mishel 2009	Examine the effects of decision-making uncertainty management intervention^a^	Theory of Uncertainty in Illness	Information about prostate cancer and communication skill	DVD + telephone + booklet, Individual + family, 7–10 days, 4 telephone calls	Nurse	Usual care
Mori 2019	Examine the effect of explicit prognostic disclosure on uncertainty^a^	N/A	Discussion about breast cancer recurrence and metastatic disease	Video, individual, N/A, 4 scripts (around 5 min)	Multiple people involved in the scripts (e.g., oncologist, palliative care physician, breast cancer survivors)	N/A
Northouse 2005	Examine the effects of a family intervention on the quality of life^a^	N/A	Information about disease, treatments; teach dyad how to be assertive to obtain additional information; help dyad learn ways to live with uncertainty	Telephone + in-person, family, 5 months, 3 home visits (90 min) + 2 phone follow-ups (30 min)	Nurse	Usual care
Northouse 2007	Examine the effects of a family intervention on appraisal variables, coping resources, symptom distress, and quality of life^a^	N/A	Information about disease, treatments; teach dyad how to be assertive to obtain additional information; help dyad learn ways to live with uncertainty	Telephone + in-person, family, 4 months, 5 sessions: 3 home visits (90 min) + 2 phone sessions (30 min)	Nurse	Usual care
Northouse 2013	Examine the effects of a brief or extensive dyadic intervention effect^a^	N/A	Information about disease, treatments; teach dyad how to be assertive to obtain additional information; help dyad learn ways to live with uncertainty	Telephone + in-person, family, 10 weeks, brief program: 3 sessions: 2 home visits (90 min) + 1 phone follow-up (30 min); extension program: 4 home visits (90 min) and 2 phone follow-ups (30 min)	Nurse	Usual care
Ritz 2000	Evaluate quality of life and cost outcomes of advanced practice nurses’ intervention	Brooten’s cost-quality model and the Oncology Nursing Society’s standards of advanced practice in oncology nursing	Information about treatment, self-care, symptom management, decision-making; care coordination such as follow-up visits, arrange multidisciplinary consults, community support groups	Telephone + in-person, individual, N/A, N/A	Nurse	Usual care
Schulman-Green 2017	Test the feasibility and acceptability of a psycho-educational intervention	Self and family management framework	Information about managing symptoms, setting goals, talking with health care providers, family and friends, managing transitions, and acting confidently	Booklet, individual, 1 month, N/A	Research staff	N/A
Sussman 2018	Test a community-based nurse-led coordination of care intervention effect^a^	N/A	Information and emotional support, and care planning	Telephone + in-person, individual, 10 weeks, 2 home visits + additional necessary phone calls and home visits	Nurse	Usual care
Tomei 2018	Test an individual cognitive-existential psychotherapy intervention effect	N/A	Psychoeducation, cognitive restructuring, behavioral activation strategies, imaginal exposure, and structured homework. The existential elements include discussion of specific fears identified through individual worst-case scenarios (e.g., death anxiety), addressing demoralization, and finding meaning in life post-diagnosis	In-person, individual, 6 weeks, 6 sessions (60–90 min)	Therapists	Delayed intervention
Victorson 2017	Examine the feasibility and preliminary efficacy of a mindfulness training program effect^a^	Mindfulness-based stress reduction	Practice of mindfulness meditation and Hatha yoga	In-person, group, 8 weeks, 8 sessions (180 min) + retreat (half day)	Mindfulness instructor	Book on mindfulness titled *Full Catastrophe Living* with no specific instructions to read it
Wang 2018	Evaluate the potential effectiveness of this mHealth supportive care intervention effect^a^	N/A	Information and communication with health providers by telephone	Phone APP, group, 3 months, N/A	1 software engineer, 1 clinical nurse, and 2 nursing researchers	Usual care
Wells-Di Gregorio 2019	Evaluate the intervention targeting a common symptom cluster in advanced cancer	Cognitive behavioral therapy; acceptance and commitment therapy	Information about interaction of thoughts, behaviors and physical tension, sympathetic arousal, stress, appraisal, coping, problem-solving, mindfulness exercise, relaxation	In-person + DVD + CD, individual, 6 weeks, 3 sessions (90 min)	Postdoctoral fellows in psychosocial oncology	Delayed intervention
Ye 2016	Examine the efficacy of a multidiscipline mentor-based program effect	N/A	Peer education and support covered illness, treatment, music therapy, traditional Chinese medicine, Taichi, and personal feelings	In-person, individual + group, 1 year, 11 sessions (180 min) + 1 group discussion	Mentor who has received training from psychologists	Usual care

^a^Study represents uncertainty management as its main aim

^b^*N/A*, data not available

#### Theoretical basis

Twelve studies (46%) described the theoretic frameworks used to guide different interventions. Five interventions were developed based on Mishel’s uncertainty in illness theory [27, 37–40]. Other theoretical models that guided the development of illness uncertainty management interventions also included the thematic counseling model [25], self-regulation theory [26], Leventhal’s common sense model [37], Brooten’s cost-quality model [41], self and family management framework [42], theory of self-efficacy, theory of stress and coping [31], cognitive behavioral therapy and acceptance and commitment therapy [43], and mindfulness-based stress reduction [44].

#### Contents and components

All of the interventions in our sampled studies included informational support that provided knowledge and resources related to illness, treatment, procedures, and symptom management. Eleven studies included emotional and psychological support from interventionist and peer groups. Nine studies included appraisal support that provided information and skills training for self-evaluation and positive perception, such as cognitive reframe and restructuring. Five studies included instrumental support that helped improve participants’ care coordination and ability to manage resources, referrals (social services, mental health, physical therapy), and continual follow-up schedules. Sixteen studies included two or more types of intervention components.

#### Mode of delivery, format, duration, and dosage

The studied interventions employed a variety of delivery modes. The majority of these interventions used both in-person and telephone delivery (*n* = 8) or in-person delivery (*n* = 7). The remaining eleven interventions used other delivery mechanisms including CD [27], DVDs [45, 46], telephone calls [38], informational booklets [42], internet [29], phone apps [36], or a combination of in-person delivery with DVD and CD content [43]. Most interventions were delivered to participants individually (*n* = 14), in a group format (*n* = 3) [36, 37, 44], or in family format (*n* = 4) [30, 32–34]. Other interventions used a combination of individual and group (*n* = 2) [25, 35], individual and family (*n* = 2) [38, 39], or group and family (*n* = 1) [31] delivery methods. The duration and dosage of the uncertainty management interventions varied across studies, ranging from one session [46] to 1-year period [35].

#### Interventionist

In thirteen studies, nurses delivered the interventions. Five interventions were delivered by professionals who had counseling or psychosocial background and training [28, 31, 35, 43, 44]. Five interventions were designed or delivered by multidisciplinary professionals [24, 34, 36, 37, 46]. One intervention was delivered by research staff [42] and one intervention design involved physicians [47]. One study did not report on the professional background of the interventionists [26].

### Intervention outcome

#### Illness uncertainty assessment

The scale most commonly used to measure an intervention’s effect on uncertainty was the Mishel Uncertainty in Illness Scale (MUIS) (*n* = 19). This scale has different versions including MUIS-Community version [48], MUIS-Survivor version [27], MUIS-Short version [40], and Parents’ Perception of Uncertainty [36], which is based on the MUIS and measures parents’ uncertainty. Other studies measured uncertainty using the symptom and ambiguity subscale of MUIS [26, 49], the Decisional Conflict Scale-uncertainty subscale [46, 47], Parent Experience of Child Illness-Short Form [34], and the Intolerance of Uncertainty Scale [43, 44]. One study measured uncertainty using a 1-item scale [24]. One study measured uncertainty using three proxy measures (i.e., problem-solving, patient–provider communication, and cancer knowledge) [39]. Most studies assessed illness uncertainty outcomes using a longitudinal design with two time points (*n* = 6); three time points (*n* = 12); four time points (*n* = 6); or six time points (*n* = 1) [41].

#### Illness uncertainty outcomes

Overall, 65% of studies (*n* = 17) suggested that an illness uncertainty management intervention had a positive effect on uncertainty outcomes. Out of the eighteen RCTs, eleven studies demonstrated that the participants in the intervention group reported significant less uncertainty than those in the control group at follow-ups. Of these studies, eight studies assessed outcomes at multiple time points. Five studies reported more reduction in uncertainty in the intervention group over time [27, 28, 35, 39, 49]. Among eight quasi-experimental studies, three studies with a control group found that participants in the intervention groups reported significantly less uncertainty compared to those in the control group [36, 40, 45]. Among five quasi-experimental studies without a control group, three studies showed that intervention participants reported a significant decrease in uncertainty over time [34, 37, 46].

### Risk of bias assessment

We evaluated each study’s risk of bias using the Cochrane Collaboration’s Risk of Bias Tool (Table 3). The eighteen RCTs had unclear (*n* = 11), high (*n* = 2), or low (*n* = 5) risk of bias. Most trials were classified as having unclear risk of bias because they did not describe the method used to generate the allocation sequence or report any strategies to maintain intervention fidelity (e.g., consistent intervention use among participants). We found that six quasi-experimental studies had high risk of bias. Most quasi-experimental studies used one-group pre- and post-designs without a control group; therefore, they had high risk of bias in random sequence generation and baseline imbalance.

**Table 3:** Assessment of study quality based on published data using Cochrane Collaboration’s criteria

Lead author and year	Random sequence generation	Allocation concealment	Blinding of participants and personnel	Blinding of outcome assessment	Incomplete outcome data	Selective reporting	Deferential intervention use	Baseline imbalance	Level of risk
Chow 2014	L	L	L	L	L	L	H	L	H
Christman 2004	U	L	L	L	L	L	U	L	U
Dharmarajan 2019	H	U	L	L	L	L	L	H	H
El-Jawahri 2010	U	L	L	L	L	L	U	L	U
Germino 2013	L	L	L	L	L	L	U	L	L
Ha 2019	H	L	L	L	L	L	U	L	H
Hendricks-Ferguson 2017	H	U	L	L	L	L	U	H	H
Kazer 2011	H	U	L	L	H	L	U	H	H
Lebel 2014	H	U	L	L	L	L	U	H	H
Liu 2006	H	U	L	L	L	L	U	H	H
McCaughan 2018	U	L	L	L	L	L	H	L	H
McCorkle 2009	U	L	L	L	L	L	U	L	U
Mishel 2002	U	L	L	L	L	L	U	L	U
Mishel 2009	U	L	L	L	L	L	U	L	U
Mori 2019	L	L	L	L	L	L	L	L	L
Northouse 2005	U	L	L	L	L	L	U	L	U
Northouse 2007	U	L	L	L	L	L	U	L	U
Northouse 2013	U	L	L	L	L	L	U	L	U
Ritz 2000	U	L	L	L	L	L	U	L	U
Schulman-Green 2017	H	U	L	L	L	L	U	H	H
Sussman 2018	L	L	L	L	L	L	U	L	L
Tomei 2018	L	L	L	L	L	L	U	L	L
Victorson 2017	L	L	L	L	U	L	U	L	U
Wang 2018	H	U	L	L	L	L	U	H	H
Wells-Di Gregorio 2019	L	L	L	L	L	L	L	L	L
Ye 2016	U	U	L	L	L	L	L	L	U

*L*, low risk; *H*, high risk; *U*, unclear

## Discussion

This study systematically reviewed the characteristics and outcomes of illness uncertainty management–related interventions for adult and childhood cancer patients as well as their family caregivers. We found that all interventions evaluated in the included studies provided informational support. Other intervention components included emotional support, appraisal support, and instrumental support. The majority of studies used both in-person and telephone or in-person intervention delivery modes. The majority of studies suggested positive effects of illness uncertainty management–related interventions on uncertainty outcomes. With only two studies focused on parents of children and adolescents with cancer, the majority of interventions were only offered to cancer patients, who were mostly older adults, female, and White.

Overall, the majority of studies (65%) found that illness uncertainty management–related interventions had positive effects on uncertainty outcomes. Multi-component interventions, which used integrated resources to target multiple aspects of illness uncertainty such as informational support and emotional support, appear to be the most effective in managing illness uncertainty in cancer patients and their family caregivers. For example, Lebel et al. found that one intervention proved effective when employing a combination of introductory material about illness, cognitive restructuring and triggers, coping skills (e.g., relaxation, calming self-talk, guided imagery), and practice expressing emotion and confronting specific fears [37]. However, the positive effects of only a few interventions appeared to endure over time, possibly indicating that many interventions’ duration should be extended or include booster sessions as needed [38].

In general, we found that uncertainty management interventions were comprised of a variety of components including informational, emotional, appraisal, and instrumental support. Informational support is the key to helping cancer patients and their family caregivers manage uncertainty. Our findings corroborate those of two previous literature reviews of psychosocial interventions for managing uncertainty in childhood cancer patients and adult patients with different chronic illnesses [14, 15]. Findings from these reviews may also collectively indicate that individualized educational interventions provide information that empowers patients to successfully develop positive coping mechanisms. These findings are consistent with core tenets of Mishel’s uncertainty in illness theory, which posits that uncertainty occurs when patients lack the information or knowledge needed to fully interpret an illness and its treatment [1]. Informational support can enlarge patients’ information and knowledge base, enabling them to better understand an illness and thus experience less uncertainty. Moreover, when uncertainty occurs, it can be difficult for patients to form a cognitive structure [1]. Appraisal supports such as cognitive reframing can help patients reinterpret their illness and view a traumatic event as manageable [38]. Emotional and instrumental support can also provide patients with psychosocial resources to manage their uncertainty.

Most interventions were delivered using either in-person and telephone or in-person formats. This finding contrasts with Zhang et al.’s systematic review and meta-analysis of patients with chronic illnesses, which identified written educational materials as the most frequently used mode of intervention delivery [15]. Given the complexity of information provision and cancer patients’ potential for psychosocial distress, in-person meetings may be the preferred mode of intervention delivery. A format combining in-person and follow-up telephone components can both evaluate patients’ current understandings of their illness and help them reassess their emotional responses [27]. Our systematic review found limited evidence of the effectiveness of technology-based (e.g., web-based, apps) uncertainty management interventions [29, 36]. This area of research is still emerging, as indicated by the recent publication dates of studies of these technology-based interventions, their pilot and feasibility research aims, and their use of small sample sizes. Given these interventions’ potential ability to provide cost-effective psychosocial services [50] to manage uncertainty across the cancer care continuum, researchers should develop and evaluate technology-based interventions for uncertainty management using a rigorous research design (e.g., RCT) with a sufficiently powered sample.

Notably, only four interventions were offered jointly to cancer patients and their spouses or partners, and only one of these reported significant improvement in the uncertainty outcome among cancer patients and their spouses [33]. This comparatively low number perhaps reflects the challenges to conducting family-based research, such as explaining the purpose of the research to multiple participants, having an extended recruitment phase that involves contacting and obtaining consent from more people, and high refusal rates [31, 51]. The small number of interventions that included spouses or partners is striking, as family caregivers play key roles in supporting cancer patients [52] and often experience higher levels of uncertainty than patients [3]. Interventions delivered to patients’ family caregivers can improve their knowledge, coping skills, and quality of life [53], which will in turn improve cancer patients’ care and outcomes (e.g., quality of life) given the synergetic interdependent relationships between cancer patients and their family caregivers [54]. There is a pressing need for future research to inform the development of interventions designed to manage uncertainty for both cancer patients and their caregivers.

Our review also indicates that future research must diversify the age, gender, and racial distributions of sample populations used to evaluate the outcomes of illness uncertainty interventions. Although previous research has shown that uncertainty is a common experience for children and adolescents with cancer [14], we identified only two interventions that assisted the parents of children with cancer to manage uncertainty [34, 36], and no intervention in our sample targeted children and adolescents with cancer. Therefore, because experiences of uncertainty can vary across different age groups or developmental stages [55], researchers should develop age-appropriate interventions that take into account the specific needs of children and adolescents with cancer. Furthermore, most of the participants in the intervention studies in this review were female, White, and older adult cancer patients. Future research regarding illness uncertainty management interventions should create strategies to increase the number of male patients and family caregivers in intervention programming. Although recruiting men for clinical trials is difficult because men are often reluctant to access services and to recognize that they need help [31], male cancer patients (e.g., prostate cancer) often experience uncertainty about their treatment decision-making and/or their management of cancer treatment-related symptoms and side effects [3, 56]. Finally, although two interventions succeed to include a sufficient number of African American cancer patients [27, 38], the majority of the study populations were White. Given that one study found that African American cancer patients reported higher levels of uncertainty than White cancer patients [3], future intervention should be tailored to help patients from minority groups and researchers should gather data about the effects of interventions using more diverse samples of cancer patients and their family caregivers.

According to the Cochrane Collaboration’s Risk of Bias Tool, most studies had “unclear” or “high risk” of bias due to their unclear reporting. Many studies have unclear reporting of the study procedures that do not meet reporting standards. Future studies should provide complete, clear, and transparent information about how to create and present a research methodology and findings in accordance with CONSORT criteria and flowchart templates.

### Limitations

This review’s findings should be considered in light of several limitations. The studies we sampled differed considerably in their study participants’ demographic variables (e.g., older, female, and White cancer patients), types of interventions, outcome measures, and timing of follow-ups. We could not conduct a meta-analysis that synthesizes their discrepant findings, which would have provided more rigorous evidence of the effects of uncertainty management interventions for cancer patients and their family caregivers. Additionally, our review only focused on interventions’ effects on uncertainty outcomes. Future research should examine the effects of uncertainty management interventions on other outcomes in order to get a more comprehensive picture of the effect of interventions. We also focused only on peer-reviewed published studies and may have missed relevant studies from the gray literature. Excluding unpublished studies likely increases the potential for biased findings; however, we included studies that reported non-significant results, thus mitigating the possibility of publication selection bias.

### Clinical and research implications

Our review has numerous implications for future clinical practice and research. Providing uncertainty management interventions with multiple components at different phases of the cancer trajectory may significantly reduce uncertainty and facilitate adaptation among patients and family caregivers. There is strong evidentiary support that multi-component interventions yield effective outcomes. However, more research is needed to compare the discrete effects of different intervention components, modes of delivery, and formats on uncertainty management outcomes among cancer patients and their family caregivers. This research should also include study populations with diverse backgrounds (e.g., by age, gender, and/or race), and in particular seek to engage children and adolescents with cancer, males, and African Americans—all groups for whom few if any tailored uncertainty management interventions currently exist.

## Conclusion

This systematic review underlines the promising potential of uncertainty management interventions—especially interventions involving multiple components including informational, emotional, appraisal, and instrumental support—to help cancer patients and their family caregivers manage illness uncertainty. Future research needs to employ a rigorous research methodology in order to test uncertainty management interventions among a diverse population and to ensure complete and accurate reporting of the research procedures and findings.

## Data Availability

All studies included in this review are publicly available.

## Appendix

**Table 4:** Cochrane Collaboration’s criteria for assessing risk of bias

Domain	Criteria	
Sequence generation	Allocation sequence was adequately generated.	Random number table Computer random number generator Coin tossing Card or envelope shuffling Throwing dice
Allocation concealment	Allocation of group assignment could not be foreseen before randomization.	Used central allocation including telephone or web-based randomization Used sequentially numbered, opaque, sealed envelopes
Blinding of participants, and personnel	Knowledge of the allocated intervention by participant and personnel was adequately prevented during the study.	No blinding but unlikely that the outcome was influenced. Blinding ensured for participants and key study personnel and unlikely to have been broken.
Blinding outcome assessment	Knowledge of the allocated interventions by outcome assessors was adequately prevented during follow-up.	No blinding of outcome assessment, but the outcome is not influenced. Blinding of outcome assessment ensured, and unlikely to have been broken.
Incomplete outcome data	Incomplete outcome data were adequately addressed.	No missing outcome data Missing outcome data unlikely related to true outcome Missing outcome data balanced across groups with similar reasons for missing data across groups Plausible effect size among missing outcomes not enough to have impact on observed effect size Missing data have been imputed using appropriate methods.
Selective reporting	The study was free of apparent selective outcome reporting.	Study protocol available and all prespecified outcomes of interest reported Study protocol is not available, but all expected prespecified outcomes reported.
Deferential intervention use	Reported outcome was among participants who similarly used interventions	All participants used intervention and complete all sessions. Adjusting the statistical analysis according intervention use
Baseline imbalance	Reported outcome was among balanced participants’ characteristics across groups.	Include all randomized participants Used stratified randomization or minimization Adjusting in the statistical analysis for baseline variables
